# Optimized culture conditions for bacterial cellulose production by *Acetobacter senegalensis* MA1

**DOI:** 10.1186/s12896-020-00639-6

**Published:** 2020-08-26

**Authors:** K. Aswini, N. O. Gopal, Sivakumar Uthandi

**Affiliations:** Biocatalysts Laboratory, Department of Agricultural Microbiology, Tamil Nadu Agricultural University, Coimbatore, Tamil Nadu 641003 India

**Keywords:** Bacterial cellulose, *Acetobacter senegalensis*, Optimization, RSM

## Abstract

**Background:**

Cellulose, the most versatile biomolecule on earth, is available in large quantities from plants. However, cellulose in plants is accompanied by other polymers like hemicellulose, lignin, and pectin. On the other hand, pure cellulose can be produced by some microorganisms, with the most active producer being *Acetobacter xylinum*. *A. senengalensis* is a gram-negative, obligate aerobic, motile coccus, isolated from Mango fruits in Senegal, capable of utilizing a variety of sugars and produce cellulose. Besides, the production is also influenced by other culture conditions. Previously, we isolated and identified *A. senengalensis* MA1, and characterized the bacterial cellulose (BC) produced.

**Results:**

The maximum cellulose production by *A. senengalensis* MA1 was pre-optimized for different parameters like carbon, nitrogen, precursor, polymer additive, pH, temperature, inoculum concentration, and incubation time. Further, the pre-optimized parameters were pooled, and the best combination was analyzed by using Central Composite Design (CCD) of Response Surface Methodology (RSM). Maximum BC production was achieved with glycerol, yeast extract, and PEG 6000 as the best carbon and nitrogen sources, and polymer additive, respectively, at 4.5 pH and an incubation temperature of 33.5 °C. Around 20% of inoculum concentration gave a high yield after 30 days of inoculation. The interactions between culture conditions optimized by CCD included alterations in the composition of the HS medium with 50 mL L^− 1^ of glycerol, 7.50 g L^− 1^ of yeast extract at pH 6.0 by incubating at a temperature of 33.5 °C along with 7.76 g L^− 1^ of PEG 6000. This gave a BC yield of wet weight as 469.83 g L^− 1^.

**Conclusion:**

The optimized conditions of growth medium resulted in enhanced production of bacterial cellulose by *A. senegalensis* MA1, which is around 20 times higher than that produced using an unoptimized HS medium. Further, the cellulose produced can be used in food and pharmaceuticals, for producing high-quality paper, wound dressing material, and nanocomposite films for food packaging.

## Background

Cellulose is a water-insoluble substance that is commonly found in plant cell walls, especially in the stalk, stem, branches, and woody parts of the plant network. The production of cellulose in nature is about 10^11^–10^12^ tons per year [1]. Cellulose, due to its abundance, has been recognized as an inexhaustible raw material to meet the demand for eco-friendly and biocompatible use [2]. The utilization of easily available raw materials such as sugarcane bagasse, banana, paddy straw, etc., can serve as an alternative source for the production of cellulose derivatives and helps to minimize deforestation [3]. On the other hand, BC is an excellent alternative to plant cellulose, which can be used for manufacturing high-end cellulose-based products [4]. BC is produced by various species of bacteria, such as *Gluconacetobacter* (formerly *Acetobacter*), *Agrobacterium*, *Aerobacter*, *Achromobacter*, *Azotobacter*, *Rhizobium*, *Sarcina* and *Salmonella*, with the most efficient producer being Gram-negative, acetic acid bacteria, *A. xylinum* [5]. Other cellulose producing bacteria can also be distinguished depending upon their source of carbon [6]. In nature, *Gluconacetobacter xylinus* forms biofilms of cellulose on the surface of fruits and flowers [7]. Intriguingly, *G. xylinus* strains, which are known to be efficient producers of bacterial cellulose, can also produce β-glucosidase [8]. A thermotolerant acetic acid bacterium, isolated in Senegal from mango fruit (*Mangifera indica*), was also found to produce cellulose [9]. Also, *Gluconacetobacter kombuchae* RG3T, isolated from Kombucha tea, displays both cellulose-producing and nitrogen-fixing characteristics [10]. The strain, *Enterobacter amnigenus* GH − 1, was subjected to various natural carbon sources like molasses, starch hydrolysate, sugar cane juice, coconut water, coconut milk, pineapple juice, orange juice, and pomegranate juice for growth and cellulose production [11]. Bacterial cellulose microfibrils from non-conventional sources such as agro-industrial residues of pineapple peel and sugar cane juice were produced by *Gluconacetobacter swingsii* [12]. Batch fermentations with the bacterial strain *Komagataeibacter sucrofermentans* using commercial sugars, and crude glycerol were also found to produce the extracellular polysaccharide [13]. The BC produced from the Egyptian *Achromobacter* sp. had pure structure without any other impurities [14].

Due to BC structure that consists of only glucose monomer, it exhibits numerous excellent properties such as unique nanostructure [15], high water holding capacity [16], a high degree of polymerization [17], high mechanical strength [12], and high crystallinity [18]. Owing to its high water holding capacity and tensile strength, microbial cellulose has become an essential raw material for products such as high fidelity acoustic speakers, papers, and dessert foods [19]. Also, BC has been used in the production of pharmaceutical and cosmetic products [20]. Nevertheless, the high production cost, primarily due to ingredients of the medium, is the major obstacle to its wide application. The critical factors affecting the BC production include the fermentation composition, i.e., carbon, nitrogen, and mineral sources used in the medium [21] and the operating conditions such as pH, temperature [22], and dissolved oxygen of the medium [23], inoculation ratio [24], and inoculum age. Though many investigators have reported BC production by various *Acetobacter* spp., the yield could not be improved to a considerable level. In order to enhance the BC yield, the present study was aimed at determining the optimum conditions viz., carbon, nitrogen, pH, temperature, precursor, polymer additives, inoculum concentration, and incubation period for achieving maximum cellulose production by *A. senegalensis* MA1. Response Surface Methodology (RSM) performed by using Central Composite Design (CCD) was used to optimize various fermentation parameters.

## Results

### Pre-optimization of culture conditions for cellulose production by *A. senegalensis* MA1

The optimum fermentation conditions, viz., carbon, nitrogen, pH, temperature, precursor and polymer additives, were investigated by measuring the wet and dry weights of the BC mats for maximum production by *A. senegalensis* MA1, and the results are discussed hereunder.

### Nutrient sources on BC production

**Fig. 1:**
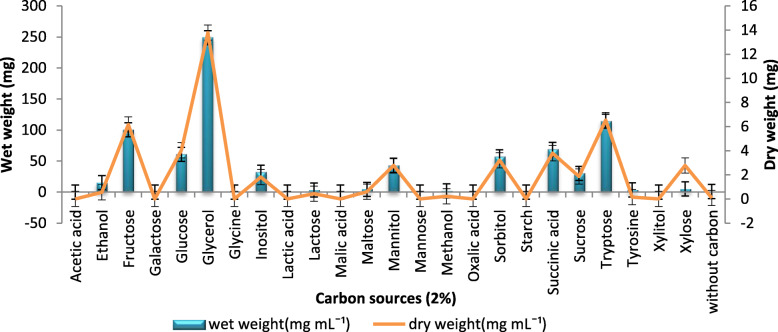
Effect of different carbon sources on BC production: BC production using different carbon sources. Rectangular bars denote the wet weight of BC produced by *A. senegalensis* MA1 and line denotes the dry weight, both provided with the error bars representing the variability of the reported measurement

BC production by *A. senegalensis* MA1 was evaluated by supplementing with 24 carbon sources, 14 nitrogen sources, different concentrations of UDP-Glucose (from 10  to 100 ppm), and 12 additives. Among the sources of carbon used, glycerol produced a maximum BC with a wet weight of 248 mg mL^− 1^, yielding a dry weight of 13 mg mL^− 1^ in HS broth (Fig. 1). This was followed by tryptose and fructose, which produced wet weight of 114 mg mL^− 1^ and 100 mg mL^− 1^ and dry weight of 6.54 mg mL^− 1^ and 6.17 mg mL^− 1^ BC, respectively. It was also observed that the carbon sources, viz., acetic acid, galactose, glycine, lactic acid, malic acid, mannose, oxalic acid, starch, and xylitol did not result in the production of cellulose. There was no significant difference in the wet weight of BC produced from glucose, mannitol, sorbitol, and succinic acid.

**Fig. 2:**
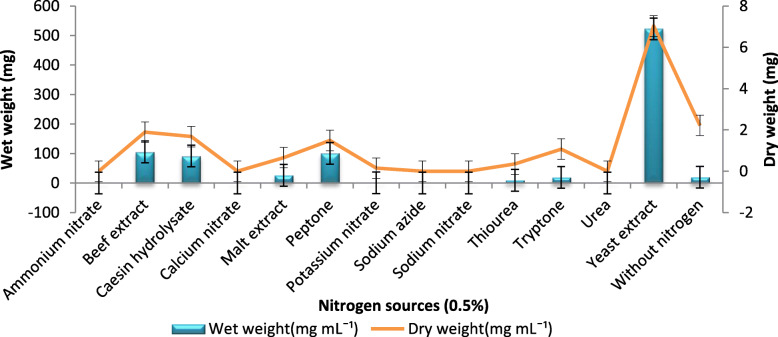
Effect of different nitrogen sources on BC production: BC production using different nitrogen sources. Rectangular bars denote the wet weight of BC produced by *A. senegalensis* MA1 and line denotes the dry weight, both provided with the error bars representing the variability of the reported measurement

Among the nitrogen sources used (at 0.5% concentration), yeast extract produced a maximum wet weight of 522 mg mL^− 1^ yielding 52.26 mg mL^− 1^ dry weight of BC in HS broth. Next to yeast extract, the beef extract produced 105 mg mL^− 1^ wet weight yielding 1.89 mg mL^− 1^ dry weight of BC followed by peptone, registering 100 mg mL^− 1^ wet and 1.49 mg mL^− 1^ dry weight. Moreover, BC production was not supported by nitrogen sources like ammonium nitrate, calcium nitrate, sodium azide, sodium nitrate, and urea (Fig. 2).

**Fig. 3:**
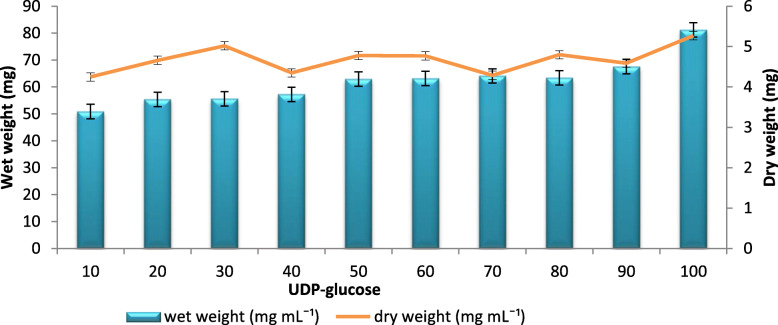
Effect of different concentrations of precursors on BC production: BC production with the addition of precursor (UDP-Glucose) to the medium. Rectangular bars denote the wet weight of BC produced by *A. senegalensis* MA1 and line denotes the dry weight, both provided with the error bars representing the variability of the reported measurement

At different concentrations of UDP-Glucose (10 to 100 ppm), the maximum wet weight and dry weight of BC (81 mg mL^− 1^ and 5.27 mg mL^− 1^ in HS broth, respectively) were produced with 100 ppm of UDP-Glucose (Fig. 3). Instead, there were no significant differences in the wet and dry weight of the BC produced from other concentrations. Since UDP-Glc is the precursor for BC biosynthesis, it was used to confirm the influence of its exogenous supply over the production.

**Fig. 4:**
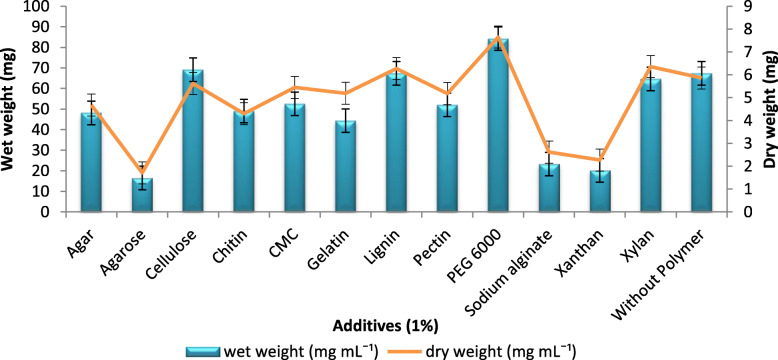
Effect of different polymer additives on BC production: BC production with polymer additives as a supplementary component. Rectangular bars denote the wet weight of BC produced by *A. senegalensis* MA1 and line denotes the dry weight, both provided with the error bars representing the variability of the reported measurement

Of the 12 different additives (at 1%) evaluated for the BC production, the addition of PEG 6000 recorded a maximum of 84 mg mL^− 1^ wet weight and 6.65 mg mL^− 1^ dry weight of BC. The addition of lignin, xylan, carboxymethylcellulose, pectin, chitin, agar, and gelatin yielded 67, 64, 52, 52, 49, 48, and 44 mg mL^− 1^ of wet BC in HS broth, respectively (Fig. 4), which was even lesser than the control that did not receive any additives. The least BC production of 16 mg mL^− 1^ wet weight and 2.51 mg mL^− 1^ dry weight was observed with the additive agarose.

### Environmental parameters on BC production

**Fig. 5:**
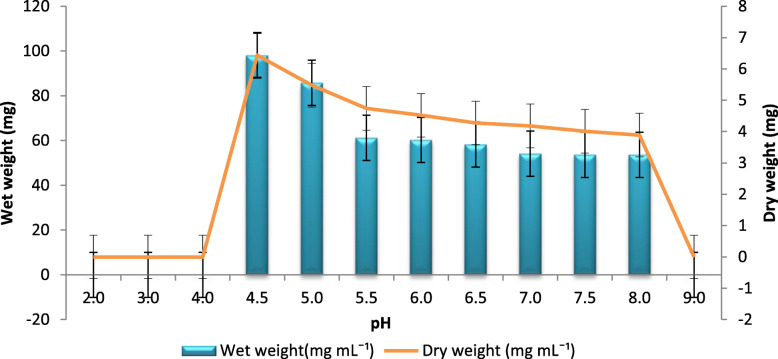
Effect of different pH values on BC production: BC production at different pH, ranging from 2.0 to 9.0. Rectangular bars denote the wet weight of BC produced by *A. senegalensis* MA1 and line denotes the dry weight, both provided with the error bars representing the variability of the reported measurement

The pH values of 2.0, 3.0, 4.0, and 9.0 showed no BC production. The maximum BC production was at pH 4.5 with a wet weight of 98 mg mL^− 1^ and a dry weight of 6.44 mg mL^− 1^, followed by pH 5.0, which produced 85 mg mL^− 1^ wet weight and 5.48 mg mL^− 1^ dry weight of BC in HS broth. At pH 5.5, wet weight of 61 mg mL^− 1^ and 4.74 mg mL^− 1^ dry weight of BC was produced (Fig. 5). Further increase in pH reduced the amount of BC production. There was no significant difference in the BC production at pH 5.5, 6.0, 6.5, 7.0, 7.5, and 8.0.

**Fig. 6:**
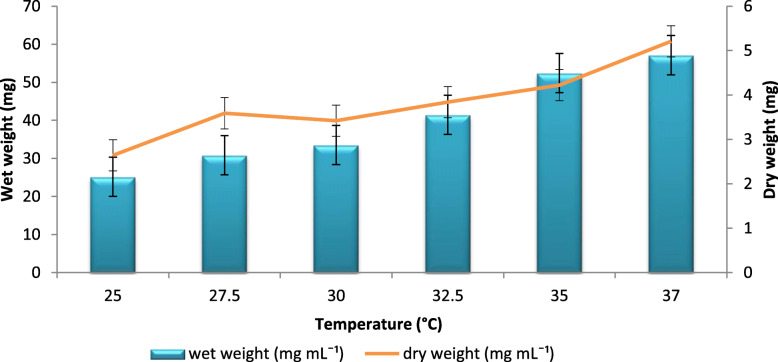
Effect of different temperatures on BC production: BC production at different temperature conditions from 25 °C to 37 °C. Rectangular bars denote the wet weight of BC produced by *A. senegalensis* MA1 and line denotes the dry weight, both provided with the error bars representing the variability of the reported measurement

Different temperatures, viz., 25 °C, 27.5 °C, 30 °C, 32.5 °C, 35 °C and 37 °C were evaluated for maximum production of BC by *A. senegalensis* MA1. The maximum BC production was observed at 37 °C of about 57 mg mL^− 1^ of wet weight and 5.21 mg mL^− 1^ of dry weight. Distinct variations in the wet and dry weight of BC were found at different temperatures. At 35 °C, the BC production was 52 mg mL^− 1^ wet and 4.22 mg mL^− 1^ dry weight. As the temperature decreases, the amount of BC produced also gets decreased at 30 °C, 27.5 °C, and 25 °C (Fig. 6).

### Inoculum concentration and incubation time on BC production

**Fig. 7:**
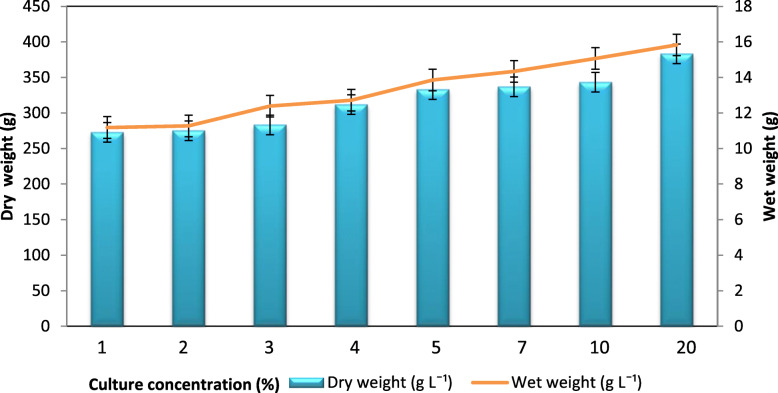
Effect of inoculum concentrations on BC production: BC production observed at varied inoculum concentrations (1 to 20%). Rectangular bars denote the wet weight of BC produced by *A. senegalensis* MA1 and line denotes the dry weight, both provided with the error bars representing the variability of the reported measurement

The highest-level of BC production was achieved with 20% of inoculum concentration, i.e.*,* at 0.545 OD, which corresponds to 395 g L^− 1^ of wet weight and 15 g L^− 1^ of dry weight. Further, at low inoculum concentration, bacterial cellulose yield was also low (Fig. 7). The concentrations of 5, 7, and 10% (0.227, 0.326, and 0.351 OD respectively) did not show a significant difference in the production of BC. The lowest production levels of 279 g L^− 1^ wet weight and 10.91 g L^− 1^ dry weight were obtained with an inoculum concentration of 1% (0.076 OD).

**Fig. 8:**
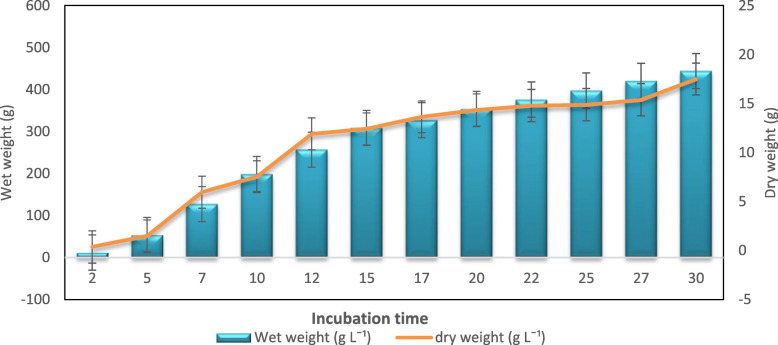
Effect of incubation time on BC production: BC production during different incubation time (2 to 30 days). Rectangular bars denote the wet weight of BC produced by *A. senegalensis* MA1 and line denotes the dry weight, both provided with the error bars representing the variability of the reported measurement

The BC production by *A. senegalensis* MA1 was evaluated by incubating for different periods, and the results implied that maximum production recorded at 30 d after incubation registering about 443 g L^− 1^ of wet weight and 17 g L^− 1^ of dry weight. The BC production gradually increased with an increase in the incubation period, and a rapid increase was found at 15 d after inoculation (Fig. 8).

### Optimized culture conditions by response surface methodology (RSM)

The culture conditions like carbon, nitrogen, pH, temperature, precursor, and polymer additives were pre-optimized and evaluated by response surface methodology using Central Composite Design (CCD). The results representing the actual and predicted values of BC production for optimization are given in Table 1. The design consists of a set of 50 runs each performed at different parameters and different levels. A quadratic design model was used for correlating the independent variables for optimization using the Design-Expert software version 11.0.5. The dependent variable contributes to different sets of independent variables. The *F* value of 3.77 for the model implies that the model is robust, and there is only 0.06% chance that such a large *F* value can occur due to noise. Likewise, the R^2^ value of 0.7221 suggested that the model is acceptable with an adequate precision of 7.5023, which indicates a desirable signal to noise ratio.

**Table 1:** Central Composite Design (CCD) matrix for the experimental and predicted values of wet BC produced by *A. senegalensis* MA1

Run Order	A: Carbon (Glycerol)	B: Nitrogen (Yeast extract)	C: pH	D: Temperature	E: Additives (PEG 6000)	Actual wet weight of BC	Predicted wet weight of BC
	**(mL L**^− 1^**)**	**(g L**^− 1^**)**		**(°C)**	**(g L**^− 1^**)**	**(g L**^− 1^**)**	**(g L**^− 1^**)**
1	50.00	7.50	6.00	33.50	1.76	387.83	343.13
2	55.00	5.00	8.00	37.00	1.00	0.00	67.06
3	55.00	10.00	8.00	37.00	3.00	0.00	19.31
4	45.00	10.00	4.00	30.00	3.00	0.00	244.17
5	50.00	7.50	6.00	33.50	3.00	440.46	378.60
6	50.00	7.50	6.00	33.50	3.00	418.89	378.60
7	61.89	7.50	6.00	33.50	3.00	370.50	185.46
8	45.00	10.00	4.00	37.00	1.00	0.00	−53.09
9	45.00	5.00	4.00	37.00	1.00	23.80	28.08
10	55.00	10.00	8.00	30.00	1.00	22.43	148.3
11	55.00	5.00	4.00	30.00	1.00	374.51	507.71
12	55.00	10.00	4.00	37.00	1.00	0.00	92.73
13	55.00	10.00	4.00	37.00	3.00	0.00	80.39
14	50.00	7.50	6.00	33.50	3.00	394.57	378.60
15	50.00	7.50	1.24	33.50	1.00	0.00	−157.43
16	45.00	10.00	4.00	37.00	1.00	0.00	41.20
17	55.00	5.00	4.00	37.00	5.00	0.00	102.59
18	45.00	5.00	4.00	37.00	1.00	0.00	8.60
19	50.00	7.50	6.00	33.50	3.00	436.37	378.60
20	45.00	10.00	8.00	37.00	1.00	0.00	76.91
21	50.0	7.50	6.00	25.18	3.00	430.41	361.36
22	45.00	5.00	4.00	37.00	1.00	0.00	8.60
23	55.00	10.00	4.00	37.00	5.00	0.00	45.14
24	45.00	5.00	4.00	30.00	5.00	415.10	265.81
25	45.00	10.00	6.00	37.00	5.00	388.63	450.91
26	55.00	5.00	8.00	30.00	5.00	0.00	182.28
27	45.00	5.00	4.00	30.00	3.00	0.00	248.71
28	45.00	10.00	4.00	30.00	1.00	334.97	92.48
29	50.00	7.50	6.00	41.82	3.00	0.00	−118.12
**30**	**50.00**	**7.50**	**6.00**	**33.50**	**7.76**	**469.83**	**432.62**
31	50.00	13.45	6.00	33.50	3.00	456.57	328.06
32	45.00	5.00	4.00	37.00	1.76	0.00	28.08
33	45.00	5.00	8.00	30.00	1.00	363.41	320.58
34	55.00	5.00	4.00	37.00	1.00	386.85	373.54
35	55.00	10.00	4.00	30.00	1.00	383.61	234.12
36	45.00	10.00	4.00	30.00	5.00	396.92	372.95
37	55.00	5.00	4.00	30.00	5.00	293.88	228.08
38	55.00	5.00	8.00	30.00	1.00	383.89	271.63
39	55.00	5.00	8.00	37.00	3.00	0.00	38.18
40	50.00	7.50	10.76	33.50	3.00	0.00	−18.73
41	45.00	5.00	8.00	37.00	1.00	97.29	105.56
42	55.00	10.00	4.00	30.00	1.00	0.00	230.50
43	50.00	7.50	6.00	33.50	3.00	378.42	378.60
44	55.00	10.00	8.00	37.00	1.76	0.00	−29.4
45	50.00	1.55	6.00	33.50	3.00	414.57	355.91
46	38.11	7.50	6.00	33.50	3.00	399.43	397.29
47	50.00	7.50	6.00	33.50	3.00	408.08	378.68
48	45.00	10.00	4.00	30.00	1.00	0.00	92.48
49	50.00	7.50	6.00	33.50	1.00	297.98	343.13
50	50.00	7.50	6.00	33.50	3.00	457.81	378.60

On the other hand, a *p* value less than 0.0500 indicates that the model terms are significant, and the results analyzed by ANOVA for the quadratic model are shown in Table 2. The model’s *F* value was calculated as a ratio of mean square regression to the mean square residual, and the *p* values were used to check the significance of coefficients, which represents the expected change in response per unit change in factor value keeping other factors constant (Table 3). The variation inflation factors (VIFs) denote how the coefficients of regression variance are inflated when the predictor variables are not linearly related, and VIFs less than 10 are tolerable. The final equation for BC production by *A. senegalensis* MA1 in terms of coded factors was given as:

**Table 2:** ANOVA for quadratic model for wet weight of BC produced by *A. senegalensis* MA1

Source	Sum of Squares	df	Mean Square	F-value	***p***-value	Significance
**Model**	1.410 × 10^6^	20	70,509.17	3.77	0.0006	Significant
A-Carbon	58,343.26	1	58,343.26	3.12	0.0886	
B-Nitrogen	991.84	1	991.84	0.053	0.8195	
C-pH	34,482.34	1	34,482.34	1.84	0.1851	
D-Temperature	2.990 × 10^5^	1	2.990 × 10^5^	15.95	0.0004	
E-Additives	45,376.76	1	45,376.76	2.42	0.1303	
AB	43,097.43	1	43,097.43	2.3	0.1442	
AC	1.753 × 10^5^	1	1.753 × 10^5^	9.34	0.0048	
AD	7017.77	1	7017.77	0.375	0.5451	
AE	1.141 × 10^5^	1	1.141 × 10^5^	6.09	0.0197	
BC	32,833.07	1	32,833.07	1.75	0.1957	
BD	84.84	1	84.84	0.0045	0.9468	
BE	47,914.04	1	47,914.04	2.56	0.1204	
CD	6716.78	1	6716.78	0.3589	0.5538	
CE	31,122.95	1	31,122.95	1.66	0.2074	
DE	77.74	1	77.74	0.0042	0.9491	
A^2^	13,140.61	1	13,140.61	0.7021	0.4089	
B^2^	2315.92	1	2315.92	0.1237	0.7276	
C^2^	3.916 × 10^5^	1	3.916 × 10^5^	20.89	0.0001	
D^2^	1.145 × 10^5^	1	1.145 × 10^5^	6.09	0.0197	
E^2^	3988.88	1	3988.88	0.2131	0.6478	

R^2^ = 0.7221, Adjusted R^2^ = 0.5304, Adeq. Precision = 7.5023

**Table 3:** Regression coefficients in terms of coded factors for optimization of cellulose production

Factor	Coefficient Estimate	df	Standard Error	VIF
Intercept	378.60	1	45.72	
A-Carbon	−44.53	1	25.22	1.47
B-Nitrogen	−5.85	1	25.42	1.50
C-pH	−36.49	1	26.88	1.54
D-Temperature	−100.81	1	25.42	1.46
E-Additives	−118.84	1	76.32	1.51
AB	−39.34	1	25.93	1.15
AC	−86.99	1	28.46	1.30
AD	16.48	1	26.92	1.24
AE	−200.23	1	81.14	1.39
BC	37.56	1	28.36	1.32
BD	−1.80	1	26.78	1.23
BE	132.81	1	83.80	1.45
CD	−17.6	1	29.38	1.43
CE	113.14	1	87.74	1.70
DE	5.16	1	80.12	1.35
A^2^	−15.42	1	18.41	1.06
B^2^	−6.47	1	18.41	1.06
C^2^	−85.58	1	18.72	1.11
D^2^	−45.43	1	18.41	1.06
E^2^	−64.79	1	140.34	1.20

Wet weight of BC = + 378.60–44.53 * A – 5.85 * B + 36.49 * C – 100.80 * D + 118.80 * E – 39.34 * (A * B) – 86.99 * (A * C) + 16.48 * (A * D) – 200.23 * (A * E) + 37.56 * (B * C) – 1.80 * (B * D) + 132.81 * (B * E) – 17.60 * (C * D) + 113.14 * (C * E) + 5.16 * (D * E) – 15.42 * A^2^–6.47 * B^2^–85.58 * C^2^–45.43 * D^2^–64.79 * E^2^.

The final equation in terms of actual factors for production of BC by *A. senegalensis* MA1 was denoted as:

Wet weight of BC = − 8134.02085 + 122.28 * Glycerol concentration + 98.89 * Yeast extract concentration + 702.13 * pH of the medium + 188.28 * Temperature of incubation + 297.57 * PEG 6000 concentration – 3.15 * (Glycerol concentration * Yeast extract concentration) – 8.70 * (Glycerol concentration * pH of the medium) + 0.94 * (Glycerol concentration * Temperature of incubation) – 8.42 * (Glycerol concentration * PEG 6000 concentration) + 7.51 * (Yeast extract concentration * pH of the medium) – 0.21 * (Yeast extract concentration * Temperature of incubation) + 11.17 * (Yeast extract concentration * PEG 6000 concentration) – 2.51 * (pH of the medium * Temperature of incubation) + 11.89 * (pH of the medium * PEG 6000 concentration) + 0.31 * (Temperature of incubation * PEG 6000 concentration) – 0.62 * (Glycerol concentration * Glycerol concentration) – 1.04 * (Yeast extract concentration * Yeast extract concentration) – 21.40 * (pH of the medium * pH of the medium) – 3.71 * (Temperature of incubation * Temperature of incubation) – 2.86 (PEG 6000 concentration * PEG 6000 concentration).

**Fig. 9:**
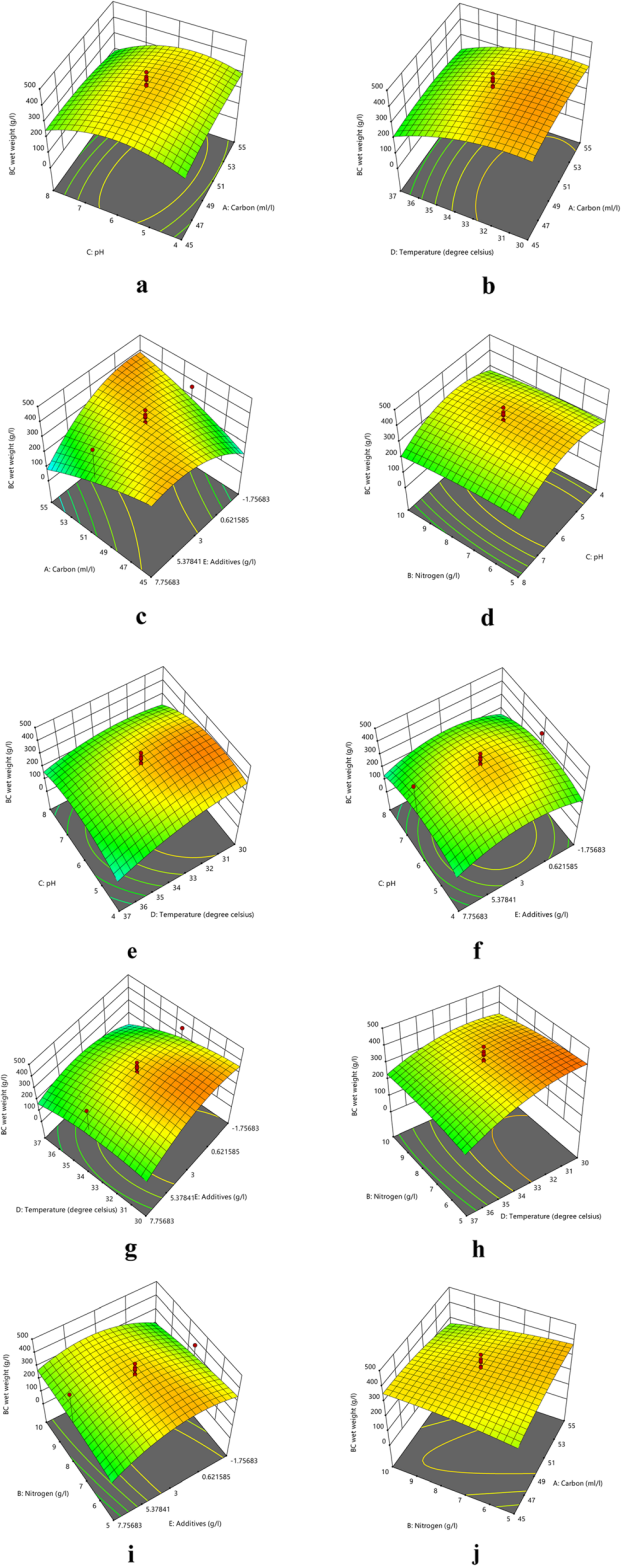
Response surface curves showing the effect of different culture conditions on BC production by *A. senegalensis* MA1: The response surface curves showing interaction between varying factors. Interactive effect is represented with the color ranging from green to red, green being lesser significant, and red being highly significant. 9a. Interaction between glycerol and pH; 9b. Interaction between glycerol and temperature; 9c. Interaction between PEG 6000 and glycerol; 9d. Interaction between yeast extract and pH; 9e. Interaction between pH and temperature; 9 f. Interaction between temperature and PEG 6000; 9 g. Interaction between yeast extract and PEG 6000; 9 h. Interaction between yeast extract and glycerol

The 3-dimensional response surface curves of different parameters are graphically represented in Fig. 9. The interaction between glycerol concentration and pH of the medium showed that the maximum production of BC could be obtained when pH is slightly acidic with 50 mL L^− 1^ of glycerol (Fig. 9a). In the interaction between glycerol concentration and temperature of incubation, the former has only a little influence compared to the latter (Fig. 9b). Further, the interaction between PEG 6000 concentration and glycerol concentration (Fig. 9c) indicates that the maximum concentration of additive (PEG 6000) along with glycerol could produce the maximum amount of cellulose. The effect of yeast extract concentration in interaction with the pH of the medium produced maximum BC at slightly acidic pH and 7.5 g L^− 1^ of yeast extract (Fig. 9d). Accordingly, the interaction of pH with temperature increased the production to a maximum at 30 °C and pH of 5.0 (Fig. 9e). The interaction of PEG 6000 concentration with the pH of the medium and incubation temperature, respectively, revealed that the concentration of PEG 6000 had a higher impact than the other two (Fig. 9f and Fig. 9g). The interaction between the temperature of incubation and yeast extract concentration indicated that maximum BC was produced at a temperature of about 30 °C with 5 g L^− 1^ of yeast extract (Fig. 9h). The effect of PEG 6000, along with yeast extract concentration, was found to be high at a medium concentration of both the sources (Fig. 9i). Also, the effect of glycerol and yeast extract concentration had no significant differences in BC production when the other factors are constant (Fig. 9j). Ultimately, the maximum production of about 469.83 g L^− 1^ of wet BC was achieved with the parameters viz., 50 mL L^− 1^ of glycerol, 7.50 g L^− 1^ of yeast extract at pH 6.0 by incubating at a temperature of 33.5 °C along with 7.76 g L^− 1^ of PEG 6000.

## Discussion

Cellulose is synthesized by bacteria belonging to the genera *Acetobacter*, *Rhizobium*, *Agrobacterium*, and *Sarcina* [25]. The most efficient producer of BC is a Gram-negative, acetic acid bacterium, *Acetobacter xylinum* (reclassified as *Gluconacetobacter xylinus*) [26, 27]. In this study, *A. senegalensis* MA1, isolated previously [28] from sugarcane juice, was used for optimizing BC production. Our earlier results demonstrated that *A. senegalensis* MA1 produced the maximum amount of cellulose mat, registering 7.2 g L^− 1^ on a dry weight basis in HS broth in 2 weeks of culturing [28]. Although the standard recommended medium for the BC production is HS medium [29], researchers have continuously attempted to optimize the media and process parameters for improving BC yields [30]. In general, a medium containing appropriate carbon and nitrogen sources is most supportive in the stable production of BC [31]. The present study optimized culture conditions such as carbon, nitrogen, pH, temperature, precursors, polymer additives, inoculum concentration, and incubation time. Although glycerol was identified as the best carbon source for maximum BC production by *A. senegalensis* MA1 in the present investigation, one of the previous studies identified mannitol as the best carbon source among the evaluated carbon sources [32]. Similarly, some experimental evidence suggests that the use of tri-carbon sugar also increased BC production [33]. Tea broth, along with a sucrose concentration of 90 g L^− 1^, resulted in 66.9% yield of BC [34].

Contrary to this, a combination of sugars such as glucose and fructose accelerated the fermentation process and maximized the yield of cellulose in lesser time [35]. Independent of the substrate used, the efficient cellulose production by the bacterium *Gluconacetobacter* sp. lies in its ability to synthesize glucose from the different carbon sources, followed by its polymerization to cellulose [36]. The synthesis of BC is a precisely and specifically regulated multi-step process that includes the synthesis of UDP-Glc, which is the cellulose precursor, followed by glucose polymerization into the β-1,4-glucan chain, and nascent chain association to form the characteristic ribbon-like structure, composed of hundreds or even thousands of individual cellulose chains [37]. In the present study, a yield enhancement in BC was seen by the incorporation of UDP-Glc as a precursor. Furthermore, depending on the carbon compound available (hexoses, glycerol, dihydroxyacetone, pyruvate, and dicarboxylic acids), the cells would begin Krebs cycle, gluconeogenesis, or pentose phosphate cycle, accounting to the production of UDP-Glc, which is a precursor for cellulose biosynthesis [38]. Several carbon compounds can be converted into cellulose with an efficiency of 50% by *A. xylinum* [39]. Supplementing the medium with ethanol instead of glucose increased the production of BC by serving as an energy source for ATP generation rather than being utilized as a carbon source [40]. On the other hand, during the utilization of glucose, gluconic acid is formed as a byproduct, which decreases the production of cellulose by reducing the pH of the culture medium. The bacterial cells cannot survive in the highly acidic medium, which hampers further production of BC. The formation of gluconic acid can be diminished in the presence of lignosulfonate [41].

Nevertheless, the microscopic and molecular characteristics of the BC remain indistinguishable with any carbon source. Evidence suggested that there is no specific pattern for a given bacterial species to utilize any desired carbon and nitrogen sources for BC production [42]. In the present investigation, yeast extract yielded maximum BC. While supplementing additional nitrogen increased biomass production but diminished the cellulose production [43]. Hence, adding nitrogen source in excess amount leads to decreased BC production, whereas the precursor molecules such as amino acids and methionine enhanced the yield [44]. The inorganic nitrogen sources in culture medium inhibited cell growth, resulting in low BC production. Whereas, the use of organic nitrogen at sufficient concentration improved the BC yield. To prevent clumping and coagulation of BC, water-soluble polysaccharides such as agar [45], acetan, and sodium alginate [46] were added as additives that enhanced the BC production in the jar-fermentor or air-lift bioreactor. The highest production of BC (8.2 g L^− 1^) was established with the addition of CMC [47]. Under static conditions, the negatively charged water-soluble cellulose derivatives, CMC, agar, and sodium alginate were used to improve the production of BC [48]. Similarly, the addition of agar at a concentration of 0.6% in the stirred-tank reactor generated 11.6 g L^− 1^ of BC by *Acetobacter xylinum* BPR2001 [49]. In the present study, addition of 1% PEG 6000 as polymer additive in HS medium enhanced the BC yield as reported earlier [28, 50].

Optimum pH is essential for oxidative reaction and normal nutrient solubility, uptake, and enhancement of BC production [51]. The pH of all media containing glucose was decreased due to the generation of gluconic acid [52]. Also, the accumulation of acetic or lactic acid in static cultures decreases the pH far lower than the optimal range required for good BC yield. The maximum cellulose yield was reported for *Acetobacter pasteurianus* HBB6 and *Acetobacter lovaniensis* HBB5 at pH 7.0 [33, 53].

The temperature plays a significant role in BC production. It has been found that *A. xylinum* culture needs a warm and static condition with the temperature not below 20 °C and not above 30 °C [54] and the ideal temperature is about 23 °C to 30 °C. The maximum production of BC was found at 30 °C by *Acetobacter pasteurianus* RSV-4 [31] and *Komagataeibacter xylinus* B-12068 [55]. In another study, an optimum temperature of 30 °C was suggested for maximum BC production, and at 45 °C, the lowest production was observed [56]. *Acetobacter* could tolerate 5 to 9% of ethanol and temperature up to 34 °C while producing vinegar [57]. The growth of *A. xylinum* 0416 at an incubation temperature of 25 °C, 27 °C, 28 °C, and 30 °C was found with no lag phase. However, at 5 °C, 20 °C, 35 °C, and 40 °C, the growth was prolonged with a more extended period of log phase [58]. A high incubation temperature can denature the bacterial cell components such as nucleic acids and proteins, even in an optimal growth medium.

If the inoculum concentration is in excess, there would be competition between the cells in utilizing nutrients, which disrupts the bacterial growth and thereby reduces the production of BC [36]. After 17–18 d of incubation, glucose in the medium was almost dissipated completely, and the metabolites reached maximum production [59]. The pellicle of BC is formed on the air-liquid interface of the media for providing sufficient oxygen to the bacterial cells. After 14 d, the pellicle attains sufficient thickness, thus restricting the entry of oxygen, which makes the cells starve for oxygen and become unable to grow actively [60]. Such operational factors can be optimized by statistical approaches such as RSM to maximize the production of BC. It can be well applied when a response or a set of responses of interest is influenced by several variables [61]. Various optimization techniques have been compared to improve the BC yields, such as one variable at a time and the design of experiments (DOE). The DOE being a statistical technique is helpful in analyzing the effect of process variables and the effects of their interactions. Plackett–Burman design (PBD) and central composite design (CCD) have been employed for BC optimization [62]. However, in the present study, the initial BC production with *A. senegalensis* MA1 was only 20.4 g L^− 1^ of wet weight and 1.02 g L^− 1^ of dry weight, which increased to approximately 20 times after 30 d of inoculation by optimizing the fermentation parameters using CCD. The results showed a correlation between the predicted and experimental responses that predicted R^2^ value of 0.7221 is in reasonable agreement with the adjusted R^2^ of 0.5304 suggesting that the model is acceptable. Whereas, in another experiment, sucrose concentration, ethanol addition, and temperature for bacterial cellulose production were optimized by RSM for *Gluconacetobacter hansenii,* and it was concluded that the addition of ethanol favors oxidation into acetic acid [63]. Taguchi method of RSM was used for optimizing the yield of bacterial cellulose and illustrated that 5% of glucose at pH 4.5 could increase the yield up to 37.5% [64]. With 10.8% sugarcane molasses and 12.5% corn steep liquor at 31 °C and pH 6.5, after 172 h, the maximum optimized production of BC by *Gluconacetobacter xylinus* C18 was attained [65]. Furthermore, the BC yield accomplished by *Komactobacter intermedius* after RSM analysis by modifying the HS medium components with 41 g L^− 1^ of fructose, and 38 g L^− 1^ of peptone was 382% higher than the standard medium [66]. *Acetobacter xylinum* produced 3.6 times more yield than the conventional one by using the fermentation condition of 29.2 °C, pH 5.83, and 1.75 g L^− 1^ glucose concentration, where 17.81 g of BC was achieved after optimization [67].

## Conclusions

Bacterial cellulose is a versatile biomaterial and has found applications in many processes giving products like high-quality paper, nanocomposites, wound repair materials, and even artificial blood vessels. Despite various applications, the cost of manufacturing of BC limits its use to a few biomedical devices and traditional fermented products, including Nata de Coco, in Asian countries. Response Surface Methodology performed by using Central Composite Design (CCD), optimized various fermentation parameters and the modification of HS medium with 50 mL L^− 1^ of glycerol, 7.50 g L^− 1^ of yeast extract at pH 6.0 by incubating at a temperature of 33.5 °C along with 7.76 g L^− 1^ of PEG 6000 for 30 days has resulted in maximum BC of 469.83 g L^− 1^. A twenty-fold higher BC yield obtained through the optimized parameters could still be enhanced through the design of a suitable bioreactor and genetic manipulation approaches. Furthermore, BC produced as such is considered as a highly pure nano polymer, any interventions of nanoformulation and extending its application toward sustainable food and pharmaceutical sector would be a much appreciable way forward.

## Methods

### Microorganisms and media

The cellulose producing bacteria, *A. senegalensis* MA1 [28] obtained from the Department of Agricultural Microbiology, Agricultural College and Research Institute, Madurai - 625,104, was previously isolated from sugarcane juice. The isolate was maintained in standard Hestrin-Schramm (HS) medium [68] composed of glucose-20 g L^− 1^, peptone- 5 g L^− 1^, yeast extract- 5 g L^− 1^, disodium hydrogen phosphate (Na_2_HPO_4_)- 2.7 g L^− 1^, citric acid- 1.15 g L^− 1^ and agar- 20 g L^− 1^.

### Pre-optimization of culture conditions for BC production

To obtain maximum BC, varying nutrients (carbon, nitrogen, precursor, and polymer additives), environment (pH and temperature), inoculum rate, and incubation period were evaluated. BC gel was produced by inoculating the freshly prepared inoculum of *A. senegalensis* MA1 (10%) in 250 mL Erlenmeyer flask containing 100 mL of sterile Modified Hestrin – Schramm (MHS) broth. After inoculation, the broth was incubated at 30 ± 1 °C for 14 d under static conditions. The mat formed at air-liquid interface was harvested and purified by alkali treatment, i.e., 2% NaOH at 80 °C for 45 min and subsequently washed with distilled water until the pH of BC was neutralized to 7.0. Prior to purification, the harvested mats were washed with deionized water. The purified mats were later dried in a hot air oven at 45 °C until BC reached constant dry weight. The concentration of peptone, Na_2_HPO_4_, and citric acid was kept constant, whereas glucose, yeast extract, pH, and temperature of the standard media varied. All the experiments were carried out under static conditions.

### Nutritional parameters

Glucose (2%), in HS medium, was replaced with other carbon sources viz.*,* acetic acid, ethanol, fructose, galactose, glucose, glycerol, glycine, inositol, lactic acid, lactose, malic acid, maltose, mannitol, mannose, methanol, oxalic acid, sorbitol, starch, succinic acid, sucrose, tryptose, tyrosine, xylitol, and xylose. Similarly, the effect of nitrogen sources viz.*,* ammonium nitrate, beef extract, casein hydrolysate, calcium nitrate, malt extract, peptone, potassium nitrate, sodium azide, sodium nitrate, thiourea, tryptone, urea, and yeast extract was evaluated by keeping other factors constant except yeast extract (0.5%). The major precursor for BC production was found to be uridine diphosphoglucose (UDP-Glc), and its effect on BC production was evaluated by different concentrations such as 10, 20, 30, 40, 50, 60, 70, 80, 90, and 100 ppm. Polymer additives such as agar, agarose, microcrystalline cellulose, chitin, carboxy methylcellulose, gelatin, lignin, pectin, polyethylene glycol (PEG) 6000, sodium alginate, and xanthan were supplemented at 1% to evaluate the maximum production of BC. A 24 h old culture of *A. senegalensis* MA1, grown for 14 d under static conditions at 30 ± 1 °C, was used for the evaluation of nutrients under all the conditions.

### Environmental parameters

HS medium prepared at different pH values of 2.0, 3.0, 4.0, 4.5, 5.0, 5.5, 6.0, 6.5, 7.0, 7.5, 8.0 and 9.0, was inoculated with 24 h old culture of *A. senegalensis* MA1 and incubated at 30 ± 2 °C for 14 d under static conditions. The effect of different temperatures on BC production was also determined by incubating at temperatures of 25 °C, 27.5 °C, 30 °C, 32.5 °C, 35 °C and 37 °C for 14 d.

### Inoculum concentrations and incubation times

Different concentrations of 24 h old *A. senegalensis* MA1 viz.*,* 1, 2, 3, 4, 5, 7, 10, and 20%, after measuring OD at 660 nm using UV-Vis Spectrophotometer (M/s. Shimadzu, Japan) were inoculated separately into 50 mL of HS broth at pH 5.0 and incubated at 30 ± 1 °C for 14 d under static conditions. The inoculum was added at the rate of 10% and then incubated at 30 ± 1 °C for different incubation periods viz., 2, 5, 7, 10, 12, 15, 17, 20, 22, 25, 27 and 30 d.

### Media optimization by response surface methodology (RSM)

RSM was used for the design of experiments for the screening of five critical media components as independent variables. The variables were glycerol as carbon source, yeast extract as a nitrogen source, pH, temperature, and PEG 6000 as a polymer additive. The dry weight of cellulose produced was fixed as the dependent variable. The design consists of 50 experimental trials, and the experiments were conducted in a randomized fashion at two levels of concentrations (high level and low level). A total of eight verification runs were performed to confirm the validity and accuracy of the model, and the experiments were done in triplicates under a static condition in 250 mL conical flasks. The natural levels and interval of variation of the independent variables in the experimental plan for the optimization of the cellulose production process are given in Table 4.

**Table 4:** Natural levels and interval of variation in the independent variables in the design of cellulose production

Factors	Codes	Levels			Interval of variation
		-1	0	+ 1	
Carbon	Glycerol (mL L^− 1^)	45.0	50.0	55.0	5.0
Nitrogen	Yeast extract (g L^− 1^)	5.0	7.5	10.0	2.5
pH		4.0	6.0	8.0	2.0
Temperature	°C	30.0	33.5	37.0	3.5
Additive	PEG 6000 (g L^− 1^)	0.0	2.5	5.0	2.5

### Statistical analysis

All the data presented in tables and figures were expressed as the mean ± standard error of three replications. Statistical analysis was done as per the method of Panse (1954) [69]. Response Surface Methodology (RSM) was employed to study the effect of different independent parameters on dependent variables using the statistical software, Design Expert (Version 11.0.5). The software was used for the data analysis, developing the regression models, and plotting the three-dimensional surface plots by employing multiple regression techniques [70]. The number of experiments designed by CCD is based on

$$ N={k}^2+2k+n $$

where *N* is the total number of experiments, *k* is the number of factors studied, and *n* is the number of replicates.

Experimental results obtained were analyzed using the response surface regression procedure of the statistical analysis system. The correlation between responses and independent variables is achieved by fitting them into the second-order polynomial equation.

$$ Y={\beta}_0+\sum \limits_{i=1}^k{\beta}_i{x}_i+\sum \limits_{i=1}^k{\beta}_{ii}{x}_{ii}^2+\sum \limits_{i=1}^k\sum \limits_{i\ne j=1}^k{\beta}_{ij}{x}_i{x}_j+\varepsilon $$

where *Y* represents the responses, *k* is the total number of independent factors, *β*_0_ is an intercept, *i*, *ii*, and *ij* with *β* represent the coefficient values for linear, quadratic, and interaction effects, respectively, and *x*_*i*_ and *x*_*j*_ in the above equation show the coded levels for independent variables.

## Data Availability

All data of this manuscript are included in the manuscript. No separate external data source is required. Any additional information required will be provided by communicating with the corresponding author via the official mail: usiva@tnau.ac.in

## Abbreviations

ATP: Adenosine Tri Phosphate
BC: Bacterial Cellulose
CCD: Central Composite Design
CMC: Carboxy Methyl Cellulose
DOE: Design of Experiments
HS: Hestrin and Schramm
OD: Optical Density
PBD: Plackett–Burman Design
PEG: Poly Ethylene Glycol
RSM: Response Surface Methodology
UDP-Glc: Uridine Di Phosphate Glucose
VIF: Variation Inflation Factors
